# Evidence of a new intermediate compound of the chitin biogenesis found in the marine-derived *Penicilliumroqueforti *fungus

**DOI:** 10.1186/1753-6561-8-S4-P212

**Published:** 2014-10-01

**Authors:** Francisco Javier Toledo Marante, Roberto Mioso, Irma Herrera Bravo de Laguna, Néstor Vicente Torres

**Affiliations:** 1 Departamento de Química, Universidad de Las Palmas de Gran Canaria, Gran Canaria, 35017, Spain; 2 Centro de Biotecnologia, Universidade Federal da Paraíba, João Pessoa, Paraíba, 58051-970, Brazil; 3 Departamento de Biología, Universidad de Las Palmas de Gran Canaria, Gran Canaria, 35017, Spain; 4 Grupo de Tecnología Bioquímica, Departamento de Bioquímica y Biología Molecular, Instituto de Tecnología Biomédica, Universidad de La Laguna, San Cristóbal de La Laguna, Tenerife, 38206, Spain

## 

Chitin derivatives, chitosan and substituted chito-oligosaccharides from fungi present a wide spectrum of applications and they have been studied in many fields such as medicine, cosmetics, agriculture, aquaculture, and food as dietary supplements [1]. Chitin is a copolymer with *N*-acetyl-D-glucosamine units linked with *β*-(1-4)-glycosidic bonds which provide rigidity to the cell wall in chitinous fungi. There is evidence that the chitin synthesis is catalyzed by the chitin synthase (CS; EC 2.4.1.16), an enzyme that transfers *β*-1,4-linked anhydro-2-acetamido-2-deoxy-D-glucose (GlcNAc) from uridine diphosphate N-acetylglucosamine (UDP-GlcNAc) to the nonreducing end of growing chitin chains [2]. However, chitin is synthesized by the regulation of distinct isoenzymes whose number ranges in some *hyphomycetes *[3]. Nevertheless, there is relatively little information on the genes responsible for chitin biosynthesis in filamentous fungi, analyses of DNA fragments from taxonomically diverse fungal species have shown that most fungi have three to six chitin synthase genes [4]. Therefore, this diversity of chitin synthases makes difficult to find a unique model of regulation of the chitin pathway. The chemical screening of the biomass of a new marine-derived strain of *Penicilliumroqueforti*, produced by liquid-state fermentation, led to the identification of several volatile and non-volatile compounds [5]. As a result of this previous study, we have isolated and characterized a new molecule. The chemical structure of the 2-deoxy-2-phosphamino-*α*-D-glucopyranose isolated was elucidated on the basis of 1D and 2D NMR studies as well as other instrumental techniques. In consequence of this discovery, it has been proposed a biogenetic route aiming to explain its formation as an intermediary component of the chitin biosynthesis.
